# Goitre Successfully Treated by Large Doses of Bromide of Potassium and Liquor Potassæ

**Published:** 1860-04

**Authors:** 


### SELECTIONS.

---

*Goitre successfully treated by large doses of Bromide of Potassium and Liquor Potassæ.*—Cases of goitre, and especially those of long standing, are some of the most troublesome and unsatisfactory that fall under the notice of practitioners. In many of them, even where treatment has been successful for a time, it sometimes happens that from very trifling exciting causes the gland enlarges to as great a size as ever. During the past year we had the opportunity of seeing over a dozen examples of goitre, under Dr. O'Conner's care at the Royal Free Hospital. In a few of those cases, iodide of potassium, in small doses, gradually increased to larger ones, with the external application of strong iodine paint, was tried without benefit. The same remedies in combination with steel, and generous living, were equally unsuccessful. Bromide of potassium, in doses of five grains, with ten minims of liquor potassæ in infusion of quassia, was next tried, and continued for some time, with benefit, the bromide being gradually increased to doses of twenty-five grains, three times a day, with forty minims of liquor potassæ. In all the instances in which it was given for a proper length of time, the result was a complete disappearance of the glandular enlargement. The minimum dose of the bromide now administered in these cases by Dr. O'Conner is ten grains, with twenty minims of liquor potassæ and infusion of quassia. There are now three patients under treatment in whom the progressively beneficial effects of this remedy are clearly discernible. In many of these cases strong iodine paint is used as an application. We have heard Dr. O'Conner state, that the bromide of potassium does not produce any of the depressing effects of the iodide, which is a practical point of very great importance.—*London Lancet.*

